# Adverse Events Related to COVID-19 Vaccines in Pregnant Women: Correspondence

**DOI:** 10.1055/s-0042-1757957

**Published:** 2022-12-29

**Authors:** Pathum Sookaromdee, Viroj Wiwanitkit

**Affiliations:** 1 Private Academic Consultant, Bangkok, Thailand; 2 Honorary Professor, Dr. DY Patil University, Pune, Maharashtra, India

Authors' reply

Dear Editor,

We would like to share ideas on the publication “Adverse Events Related to COVID-19 Vaccines Reported in Pregnant Women in Brazil.” 1 According to Kobayashi et al., 1 ten deaths were identified, one of which was thought to be causally related to the vaccine, and the other nine maternal deaths had causality C, that is, no causal relationship with the vaccine, and the majority were due to complications inherent in pregnancy, such as pregnancy-specific hypertensive disorder (PSHD) in four cases, and COVID-19 in three. 1 The problem of the efficacy and safety of the COVID-19 vaccine for pregnant women is intriguing, and evidence from real-world situations are needed. 2 Aside from death, there are further concerns about the safety of the COVID-19 vaccine for pregnant women, including the induction of abortion. 3

We are all concerned that, despite its benefits, the COVID-19 vaccine may be harmful. It is difficult to determine the exact source of the clinical issue in this case due to a lack of information on the health and immunological status of vaccination recipients prior to inoculation. People may reject vaccines and lose faith in them if they are given contradicting information. A clinical comorbidity may be at the basis of the problem. 4 5 The assessment of potential detrimental outcomes is now too early due to incomplete documentation of a person's health or immune condition prior to immunization.
